# An Overview of Monthly Rhythms and Clocks

**DOI:** 10.3389/fneur.2017.00189

**Published:** 2017-05-12

**Authors:** Florian Raible, Hiroki Takekata, Kristin Tessmar-Raible

**Affiliations:** ^1^Max Perutz Laboratories, University of Vienna, Vienna Biocenter, Vienna, Austria; ^2^Research Platform “Rhythms of Life”, University of Vienna, Vienna Biocenter, Vienna, Austria

**Keywords:** circalunar, circadian, moon, light, sleep, mood, marine, reproduction

## Abstract

Organisms have evolved to cope with geophysical cycles of different period lengths. In this review, we focus on the adaptations of animals to the lunar cycle, specifically, on the occurrence of biological rhythms with monthly (circalunar) or semi-monthly (circasemilunar) period lengths. Systematic experimental investigation, starting in the early twentieth century, has allowed scientists to distinguish between mythological belief and scientific facts concerning the influence of the lunar cycle on animals. These studies revealed that marine animals of various taxa exhibit circalunar or circasemilunar reproductive rhythms. Some of these rely on endogenous oscillators (circalunar or circasemilunar clocks), whereas others are directly driven by external cues, such as the changes in nocturnal illuminance. We review current insight in the molecular and cellular mechanisms involved in circalunar rhythms, focusing on recent work in corals, annelid worms, midges, and fishes. In several of these model systems, the transcript levels of some core circadian clock genes are affected by both light and endogenous circalunar oscillations. How these and other molecular changes relate to the changes in physiology or behavior over the lunar cycle remains to be determined. We further review the possible relevance of circalunar rhythms for terrestrial species, with a particular focus on mammalian reproduction. Studies on circalunar rhythms of conception or birth rates extend to humans, where the lunar cycle was suggested to also affect sleep and mental health. While these reports remain controversial, factors like the increase in “light pollution” by artificial light might contribute to discrepancies between studies. We finally discuss the existence of circalunar oscillations in mammalian physiology. We speculate that these oscillations could be the remnant of ancient circalunar oscillators that were secondarily uncoupled from a natural entrainment mechanism, but still maintained relevance for structuring the timing of reproduction or physiology. The analysis and comparison of circalunar rhythms and clocks are currently challenging due to the heterogeneity of samples concerning species diversity, environmental conditions, and chronobiological conditions. We suggest that future research will benefit from the development of standardized experimental paradigms, and common principles for recording and reporting environmental conditions, especially light spectra and intensities.

## The Occurrence of Circalunar Rhythms and Clocks

Physiological processes and behaviors often occur at specific times. Similar to human societies that follow not only the pace of the watch but also that of the calendar, many organisms structure their behavior and physiology not only by the regular cycles generated by the changes of sun (daily and seasonal timing) but also the cycles of the moon (monthly timing). Moreover, different timing regimes can also be used in combination, for instance, to synchronizing reproduction to a particular season of the year, particular day(s) of the month and specific hours during these days.

**Figure 1:**
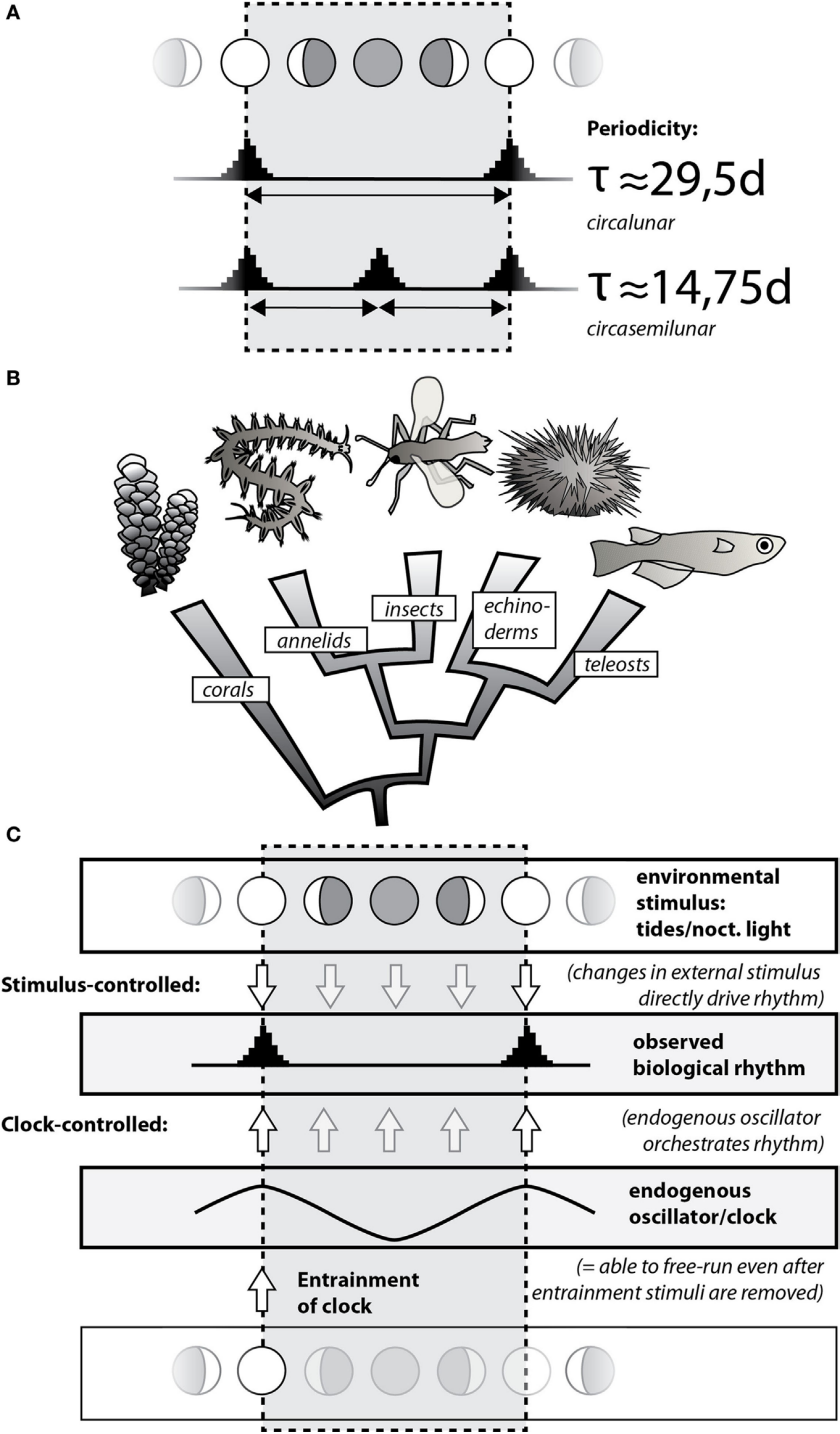
**Circalunar and circasemilunar rhythms and clocks/oscillators are widely present in the animal kingdom**. **(A)** Common biological rhythms linked to the moon cycle can be classified into circalunar and circasemilunar rhythms based on their periodicity, reflecting the re-occurrence of specific events/states once or twice, respectively, during the lunar month. Note that these events/states can be matched with any of the lunar phases, with the example showing synchrony with the full/new moon. **(B)** Circalunar/circasemilunar rhythms are found in a broad range of animals, as demonstrated by the phylogenetic position of individual animal groups in which reproductive cycles have been linked to the lunar phase (see text). **(C)** Biological rhythms either reflect direct response of an organism to changes in the respective environmental stimulus, such as nocturnal light (top; “Stimulus-controlled”); or they are driven by endogenous clocks that are entrained/set by a particular state of the environmental stimulus (bottom; “Clock-controlled”). As the environmental stimulus is not required for an endogenous clock to continue, a clock-mediated biological rhythm also “free-runs” if the environmental stimulus is experimentally removed.

Generally, periodic organismal processes (biological rhythms) can be orchestrated in two different ways (Figure 1A). On the one hand, they may be generated directly by changes in the regular external cues. Such a setting allows a given rhythm to adjust rapidly to any sudden changes in the external cues, but in turn makes the rhythm inherently sensitive to disturbance. On the other hand, organisms can possess internal timing systems of the respective period length (so-called biological clocks) that are adjusted to the external cues, but able to continue to run independently, thereby making the biological rhythm more robust against short-term disturbances. The evolution of such biological clocks has likely been favored by the extreme stability of geophysical cycles and the advantages organisms have when they can not only react to regular changes in the environment but also anticipate these changes and prepare accordingly.

Whereas biological rhythms have been observed over centuries, molecular details have so far best been worked out for the biological rhythms and clocks running on a 24-h cycle, reflecting the day and night cycle. Over recent years, progress has also been made in the molecular understanding of seasonal rhythms. Both rhythms reflect the natural cycles of the sun. This review focuses on rhythms and clocks of period lengths provided by the moon. These run with around 29.5 days (circalunar rhythms/clocks) or 14.75 days (circasemilunar rhythms/clocks) (see Figure 1A). The moon also generates rhythms with shorter period length of 12.4 and 24.8 h, so-called circatidal and circalunidian rhythms, respectively (1–5). We do not discuss these rhythms in our review, because they cover a time scale that is very different from the monthly and semi-monthly rhythms, and are thus likely to be functionally distinct.

Circalunar and circasemilunar rhythms are widespread among organisms, especially in the context of reproductive cycles of marine animals. This fact was likely already noted by fishermen in antiquity, due to the practical implication that the size of the (edible) gonads of local sea urchins varied over the lunar month (6). The notion became a piece of cultural memory through its generalization in Aristotle’s work (De partibus animalium IV, 5), and its further tradition by classical authors [see Ref. (6) for the historical reception of the concept]. In the 1920s, the British zoologist Harold Munro Fox put the classical statements to systematic scientific tests, confirming the observation of lunar phase-dependent gonad changes in the Egyptian sea urchin *Diadema setosus*, while dismissing the concept for several other species (6, 7). Fox and other researchers (6–8) also started to compile published evidence for circalunar and circasemilunar rhythms in other marine species, a list that has steadily grown over the course of subsequent decades (2). Figure 1B provides some of the well-established examples for circalunar reproductive cycles in marine animals: the seasonal spawning of tropical corals such as *Acropora* during full moon nights (9), the reproduction of the annelid worm *Platynereis* during the waxing moon (10, 11), the precise emergence of the midge *Clunio* at neap tides (12), the lunar cycles of gonad growth in the sea urchin *D. setosus* (6, 7), as well as the circalunar spawning of several fish species (13), such as the goldlined spinefoot (*Siganus guttatus*) in tropical reefs (14), the California grunion (*Leuresthes tenuis*) (15), or the mummichog (*Fundulus heteroclitus*) (16). Besides its impact on reproductive cycles, the lunar cycle also affects the behavior of marine animals. For instance, during the Arctic winter, massive waves of diel vertical migration of the zooplankton are linked to the lunar cycle, reflecting the importance of moonlight as the predominant light stimulus in that period (17). While our review focuses on the animal kingdom, it should be noted that circalunar or circasemilunar reproductive rhythms also exist in species of other eukaryotic kingdoms, such as the brown alga *Dictyota dichotoma* (kingdom Chromalveolata) (18) or the Peruvian apple cactus *Cereus peruvianus* (kingdom Archaeplastida) (19, 20).

The aforementioned distinction between externally regulated rhythms and clock-mediated rhythms is also relevant for the discussion on the occurrence of circalunar rhythms. On the one hand, a reliable, monthly fluctuating environmental stimulus—such as the light stimulus of the full moon, or the mechanical stimulus of the spring/neap tides—can directly cause variation in animal physiology, pigmentation, or behavior, or trigger subsequent hormonal changes. In each of these cases, the stimulus directly translates into an observable biological rhythm (schematized in Figure 1C as “Stimulus-controlled”). On the other hand, the respective stimulus can also act to entrain a circalunar timing mechanism (a circalunar clock, also referred to as “circalunar oscillator” in this review). This clock then drives the observed circalunar rhythm (Figure 1C, “Clock-controlled”). A classical experimental approach in chronobiology that distinguishes between these two possibilities is the omission of the stimulus after an initial “entrainment” phase (Figure 1C, “Entrainment”) [see, e.g., Ref. (21)]. Whereas a circalunar rhythm produced by direct impact will not persist under such conditions, a clock-mediated circalunar rhythm will be able to persist. Currently, nomenclature for such omission experiments differs [e.g., “free-running full moon” (22); “constant new moon” (23)].

## Approaches to Unravel the Molecular and Cellular Mechanisms of Circalunar Rhythms and Clocks in Marine Systems

Even though circalunar and circasemilunar rhythms and clocks are widespread, and common in the marine environment, researchers have only recently started to tackle the underlying molecular and cellular changes and mechanisms. Most of the molecular data focus so far on the analysis of known circadian clock genes, putative photoreceptors, as well as transcriptomic studies over the course of circalunar rhythms (see Table 1). It lies in the nature of these approaches that most of the results are still on the correlative level. Here, we provide an overview of a selection of recent molecular approaches and try to derive more general conclusions from these studies.

**Table 1:** **Overview on gene differences in the context of the lunar cycle**.

Species	Genes analyzed for being affected by nocturnal light or circalunar clock	Analytical method(s)	Reference
*Acropora millipora* (coral)	cry1, cry2 expression at noon vs. midnight during new moon and full moon, protein location in tissue (note that coral cry1 and cry2 are not equivalent to bilaterian cry1/cry2)	qPCR	Levy et al. (24)

*A. millipora* (coral)	cry1, cry2, clk, cycle, tim, eya expression 2 sampling regimes: – every 4 h during new and full moon – midnight on 4 moon phases and 4 different lunar light regimes (normal lunar cycles, constant new moon, constant full moon)	qPCR	Brady et al. (23)

*A. millipora* (coral)	Transcriptome from various diel and lunar timepoints	Quantitative RNAseq	Kaniewska et al. (25)

*Acropora gemmifera* (coral)	Transcriptome from two diel and four lunar timepoints	Quantitative RNAseq	Oldach et al. (26)

*Favia fragum* (coral)	*cry1, cry2, clk, cycle* expression at various diel and lunar timepoints	qPCR	Hoadley et al. (27)

*Platynereis dumerilii* (annelid worm)	*clock, bmal (cycle), tr-cry, L-cry, period, pdp1, vrille*	qPCR	Zantke et al. (22), Tessmar-Raible et al. (28)

*Clunio marinus* (dipteran insect)	Genomic loci that contain the genetic differences causing differences in monthly timing	QTL mapping/genome sequencing	Kaiser et al. (29)

*Siganus guttatus* (fish)	*cry1, cry3, per4* transcripts at noon during 5 different lunar phases (natural lunar light, constant new moon, two different coverage regimes during different phases of the night)	qPCR	Fukushiro et al. (30), Toda et al. (31)
	Cry3 protein localization in brain		

### Characterization of Molecular and Behavioral Impacts of Circalunar Rhythms and Clocks

#### The Relationship of Circadian and Circalunar Rhythms

As mentioned earlier, circalunar timing mechanisms rarely exist in isolation, but are coordinated with other timing mechanisms, such as daily (circadian) timing. Therefore, several studies have investigated if the expression of known circadian clock genes is affected either by nocturnal light or by the phase of a circalunar clock (Table 1).

In this context, the genes encoding members of the Cryptochrome (Cry) family have received particular attention. Crys are flavoproteins involved in cellular signaling, which are anciently related to photolyases, UV-responsive DNA repair enzymes (32). Molecular phylogenetic analyses show that Crys form multiple, evolutionarily conserved subgroups (32–34). Several of these subgroups are of interest for circadian clock research: members of the d-Cry/Cry1/Lcry family can function as photoreceptors (activated by short-wavelength light) in insect and annelid circadian clocks (22, 33, 35, 36), whereas members of the distinct v-Cry/Cry2/tr-Cry family function as transcriptional repressors in the circadian transcription/translational core loop [reviewed in Ref. (37)]. Members of a family called “plant-type Cryptochromes” also exist in animals and diatoms. In plants, members of this family have been shown to function as photoreceptors for the plant circadian clock [reviewed in Ref. (38)]. Their role outside of the plant phylum is currently unknown (34).

Cryptochrome genes have been investigated in several coral species that display lunar reproductive cycles. In the coral *Acropora millepora*, three types of Crys were identified (24): *Ami-*Cry1 has closest homology to tr-Crys, *Ami-*Cry2 is positioned in the group of the 6-4 photolyases, while the third molecule, *Ami-*CryDash, is related to another ancient group of Crys that has been dubbed “cry-*Drosophila, Arabidopsis, Synechocystis, Homo*” (Cry-DASH) due to its broad evolutionary conservation (32). Different studies investigating the mRNA expression levels of *cry1* and *cry2* and their possible modulation in lunar reproductive cycles arrived at different results: the first study by Levy et al. showed that both *cry1* and *cry2* are induced by sunlight (with no reproducible transcript changes without light). In addition, when animals were sampled during natural full moon nights, *cry2* transcript levels were significantly higher than during new moon nights (24), while *cry1* levels did not show a difference. These results contrast with more recent research by Brady and co-workers in the same coral (23). While these researchers also describe changes in transcript levels for the *cry2* gene over the lunar light cycle, *cry2* showed elevated expression levels at midnight during new moon—and not full moon—nights. Furthermore, in their study, also *cry1* levels showed fluctuations, with elevated expression levels at midnight during the first-quarter moon (23). This study also tested transcript oscillations under constant nocturnal light and lack of nocturnal light over the course of an entire lunar cycle. These experiments assessed if the transcriptional changes are under the control of an endogenous oscillator or only under direct light control. Interestingly, midnight *cry1* and *cry2* transcript levels still showed differences at different phases of the lunar cycle independent of illumination, consistent with the idea that corals also possess a circalunar clock.

Finally, changes in gene expression in *A. millepora* over the lunar month have also been assessed using a transcriptomic approach (25) (also see below). In this study, *cry1* transcript levels were highest at midnight during full moon nights (25). The cause of these differences is currently unclear and could range from variants in the environmental conditions or different subspecies to higher variation in the transcript changes than previously anticipated. Transcript levels of *cry1* and *cry2* orthologs have also been analyzed in a different coral species, *Favia fragum*. Both genes exhibit light-controlled daily oscillations and also transcript level differences between different moon phases (27). The correlation between the moon phase and the transcript level is, however, not fully clear, since no full lunar cycle was analyzed. Taken together, despite several discrepancies, these results suggest that *cryptochromes* are interesting genes for studying the effect of the lunar cycle on corals, and possibly allowing conclusions on the impact of the lunar cycle on circadian biology of these animals. However, as corals branch off the animal tree at a very basal position, one restriction at this point is that it is still unclear which of the investigated Crys are functionally relevant for circadian control in corals.

The assignment of Crys to circadian functions might be less problematic in other taxa, where Crys have also been investigated, given the clearer functional subgroup position. In the golden rabbit fish, *S. guttatus*, mRNA levels of two *tr-cry* homologs—*SgCry1* and *SgCry3*—fluctuate with the lunar cycle in the brain, but not the ovary (30). Whereas *SgCry1* levels are controlled by light, *SgCry3* levels continue to exhibit a monthly periodicity even in the absence of nocturnal light cues, providing strong evidence that this gene is under the control of an endogenous monthly clock (31).

The bristle worm *Platynereis dumerilii* possesses a complete set of animal Cry/photolyase genes, with one ortholog for each distinct subfamily (34). Of those, *tr-Cry* and *L-Cry* have been investigated with respect to nocturnal light cycles and the circalunar clock of the worm (22). When tested in S2 tissue culture cells, *Platynereis* tr-Cry functions as a transcriptional repressor, but not a light receptor, consistent with a conserved function of this molecule in transcriptional circadian control. The transcripts of *tr-cry* show a clear circadian rhythmicity (both during circadian light–dark and dark–dark conditions). Under nocturnal light conditions that are sufficient to reset the circalunar clock of these animals, the oscillations of *tr-cry* are abolished, indicating that nocturnal light stimuli can affect circadian clock gene expression. Conversely, there is no significant effect of the lunar clock itself on the transcript levels of this gene (22, 28). Also, *Platynereis L-cry*, which functions as a light receptor when tested in S2 cells, shows fluctuations in transcript levels between day and night. These, however, do not appear to follow a regular circadian pattern (22). Both nocturnal light and the circalunar clock appear to impact on the expression of this gene. Due to the irregularity of *L-cry* regulation, however, these changes are difficult to quantify reliably [Ref. (22, 28); Zantke and Tessmar-Raible, unpublished observations].

Besides *cry* genes, also other circadian clock gene homologs have been studied in these animals. In the bristle worm *Platynereis*, transcript levels of the core circadian clock genes, *pdp1, period*, and *clock*, exhibit clear changes depending on the worm’s endogenous circalunar clock: compared to samples taken during new moon phase, levels are significantly elevated during the full moon phase, even in the absence of nocturnal light (“free-running full moon”) (22). Interestingly, a circalunar regulation that persists under free-running conditions has also been observed for transcript levels of *per4* in the diencephalon of the reef fish *S. guttatus*. In the brain samples that were taken at different timed during the lunar cycle, this gene had its lowest expression around the first quarter of the moon, even if the fish were shielded from light during the night (31). Finally, free-running regulation was also observed for several coral genes, like the presumptive circadian clock genes *Ami-cycle, Ami-clock*, and *Ami-tim* (23).

Taken together, it appears that both natural and experimental changes in nocturnal illumination, as well as endogenously running circalunar clocks impact on the transcript levels of circadian clock gene homologs in marine organisms as diverse as corals, annelid worm, and fish. A major task for the future will be to work out if and how these transcript changes impact on the circadian rhythm of the respective model species. Interestingly, at least in the bristle worm *P. dumerilii*, the circalunar clock has also been shown to impact on circadian rhythms of locomotor activity, suggesting the possibility that the observed transcript regulations might be linked to these activity changes (22). Such behavioral changes might be due to hormonal fluctuations, as it has been shown that in several species with lunar controlled reproductive cycles, hormones, and hormonal receptors change with the lunar light cycle. In vertebrates, the melatonin pathway is one of the hormone pathways affected by the lunar cycle. For instance, moonlight changes the abundance of *aanat1* (the precursor of the synthesis enzyme AANAT) in the eye of the goldlined spinefoot *S. guttatus* (39). Moreover, at least two of the melatonin receptors in the mudskipper, *Boleophthalmus pectinirostris* fluctuate with semilunar periodicity, in phase with the *aanat2* gene in the pineal of that species (40). Such results provide interesting entry points for further research into the question how nocturnal light modulates circadian biology of animals. In turn, another conceptually interesting question is if the circadian clock components themselves are involved in the generation of circalunar or circasemilunar rhythms. Pharmacological interference experiments in the bristle worm *P. dumerilii* suggest that circadian clock gene oscillations are not required to maintain circalunar rhythms in this species (22). But this does not exclude a role for the circadian clock in entraining the circalunar clock (also see discussion below). Moreover, mass spawnings of marine animals are often not only synchronized to particular days but also particular hours of the day—sometimes with extreme precision (8, 9, 15). Such cases would predict that circadian and circalunar clocks are likely to converge at least on the level of regulating mating behavior or gamete release. Research into the interaction of circadian and circalunar clocks may therefore reveal interesting insight into the coordination between distinct timing mechanisms.

#### Omics Approaches to Identify Fluctuations Correlated with the Lunar Cycle

Whereas the aforementioned work investigated specific effects of the lunar cycle on circadian clock components, several researchers have tried to complement these experiments with broader approaches that also explore possible rhythmicity in the expression of other genes. High-throughput transcriptome profiling has become an attractive technology for this research. Again, work on corals has already spearheaded this direction (Table 1). Quantitative RNA sequencing was performed on two *Acropora* species over the course of the lunar cycle. Samples taken from *Acropora millipora* at three different times during the day on new moon vs. full moon days revealed that 2% (midnight) to 6% (noon) of *Acropora* genes fluctuate between the two lunar conditions. Based on functional annotation of the encoded proteins, the regulated genes cover a variety of different biological processes, including cell communication, cell differentiation, and cell proliferation (25).

In the second study, *Acropora gemmifera* branches were sampled at four different moon phases and during two different times of the day (noon and midnight). Two sets of regulated transcript types were identified from the quantitative RNA sequencing: one set (55 isogroups) showed diurnal expression patterns that fluctuated over the course of the lunar cycle, whereas the second set (273 isogroups) exhibited differential expression over the lunar cycle when noon and midnight sampling timepoints were combined (26). These two gene sets were largely non-overlapping, resulting in an overall detected change of transcripts over the lunar cycle of about 0.6% [Ref. (28); Vince, personal communication concerning which EST dataset was exactly used for the mapping of the reads]. When considering these numbers, it should be noted, however, that in the second study, sequencing reads were mapped across species (i.e., *A. gemmifera* RNAseq reads onto an *A. millipora* transcriptome). It is thus likely that the real number of regulated transcripts is higher, since genes with lower sequence conservation would not map reliably.

More such studies, especially also under free-running conditions, will be needed to understand the impact of nocturnal light and the circalunar clock on the transcriptome of animals. One challenge that is already emerging from the data reviewed above is that experimental design, data acquisition, and analysis methods differ between studies, making comparisons between individual experiments difficult. A general trend in all of the reviewed studies is that both nocturnal light and free-running circalunar clocks impact on the transcript level of specific genes. The extent of this phenomenon, as well as the potential conservation of such regulated transcripts, remains to be analyzed in the future. Also, the functional meaning of such transcript changes is currently unclear.

#### Forward Genetic Approaches to Identify Molecules That Can Modulate Circalunar Timing

The third approach to identify molecular mechanisms involved in circalunar timing is to investigate factors that modulate this timing mechanism in natural populations. This approach draws on the idea that within the population of a given species, individual differences in timing exist. In humans, and with reference to daily timekeeping, such natural variants are called chronotypes, with the extremes of “larks” (early chronotypes) and “owls” (late chronotypes) (41). Individual timing differences, however, are neither restricted to humans nor to differences in the circadian clock. One very attractive model system is the non-biting marine midge *Clunio marinus*. This species possesses chronotypes with respect to both daily and monthly timing mechanisms, thereby allowing individual populations to time their emergence precisely to the local neap and spring tides (42). Importantly, these timing differences were shown to be genetically inherited (43). Combining rigorous genetic mapping of these differences with a high-resolution genome for this insect, as well as re-sequencing of distinct chronotypes, has recently allowed the identification of several candidate genes modulating circalunar (and circadian) timing in distinct *C. marinus* timing strains (29). Interestingly, the current analysis in the midge is consistent with the idea that circadian and circalunar timing mechanisms are distinct, as none of the core circadian clock genes is involved in circalunar timing variation (29). It is, however, currently still unclear if the gene loci responsible for the circalunar “chronotypes” are relevant for the entrainment pathway, the circalunar clock, or the output pathway. Hence, it can at presence not be excluded that there is an overlap between some components relevant for circadian and circalunar timing in this insect. The hope is that functional experiments in the midge will help to unravel by which mechanism any of the current candidate loci really contributes to the fine tuning of circalunar timing.

### The Quest for the Moon Light Sensors

Another central question concerns the identity of the light receptor(s) that allow organisms with light-driven circalunar rhythmicity to perceive dim nocturnal light, and thereby endow these species either with the ability to directly react to nocturnal light or—in species where circalunar clocks exist—entrain these clocks to the light stimulus.

Due to their light-responsive properties, Crys—that we have discussed above in the framework of the circadian clock—have also received significant attention in this context. The aforementioned study by Levy in the coral *A. millepora* was the first to propose a member of the Cryptochrome family (Cry2) as a possible moon light sensor that could impact on the mass spawning of the coral around full moon (24), and thereby nurtured further interest in this protein group in other studies of animals displaying circalunar rhythms, including the research into reef fish discussed earlier (30).

While such studies suggest a possible function of Cry molecules—albeit of distinct subgroups—as nocturnal light receptors, it is important to emphasize that the speculation on the function of these molecules currently still relies on correlation between the regulation of transcript levels and environmental light. A functional requirement for moon light reception has neither been demonstrated for the reef fish nor for any of the corals. It is also unclear if upregulation of the respective genes is correlated with enhanced light receptive function, i.e., if the mRNA regulation translates into levels of functional protein. Of note, for the coral, the same authors have recently suggested another class of photoreceptors—a melanopsin—as possible light receptor relevant for gamete release, also based on RNA expression data (25).

Opsins have also been suggested to play a role in moonlight sensation in other models. A peculiar example is the Somalian cavefish *Phreatichthys andruzzii*. This species inhabits the dark phreatic layers beneath the desert and has evolved in isolation from surface populations for an estimated time of 3 million years (44). Whereas the species has lost its eyes, as well as many of its photoreceptor genes (45, 46), several Opsins have remained fully functional (47). Together with observations that fish swim up to the surface of oasis fountains during moonlit nights, this has led to the speculation that these Opsins could be involved in moonlight reception [Ref. (47); Bertolucci, personal communication].

Given these different proposals, a key task for the future will be the functional test of individual light receptors in suitable model species. Here, an attractive model species in which the relevance of Opsins and/or Cryptochrome family members for lunar light reception is being tested is the marine bristle worm *P. dumerilii*. A classical study tested by tissue ablation if the worm’s eyes are required for circalunar entrainment, concluding that the adult eyes are dispensable for that purpose (48). More recently, transgenesis and genome mutagenesis have allowed the generation of knockout strains for specific genes (49). A mutant strain has already been generated for the *L-cry* ortholog of the bristle worm, which is currently being used to test the contribution of this gene to circalunar entrainment (49). Interestingly, previous analyses on the properties of the light suitable for the entrainment of the worm’s circalunar clock suggests that nocturnal light of different wavelengths is suitable as entraining stimulus (11). This may indicate the involvement of more than one photoreceptor in this process.

**Figure 2:**
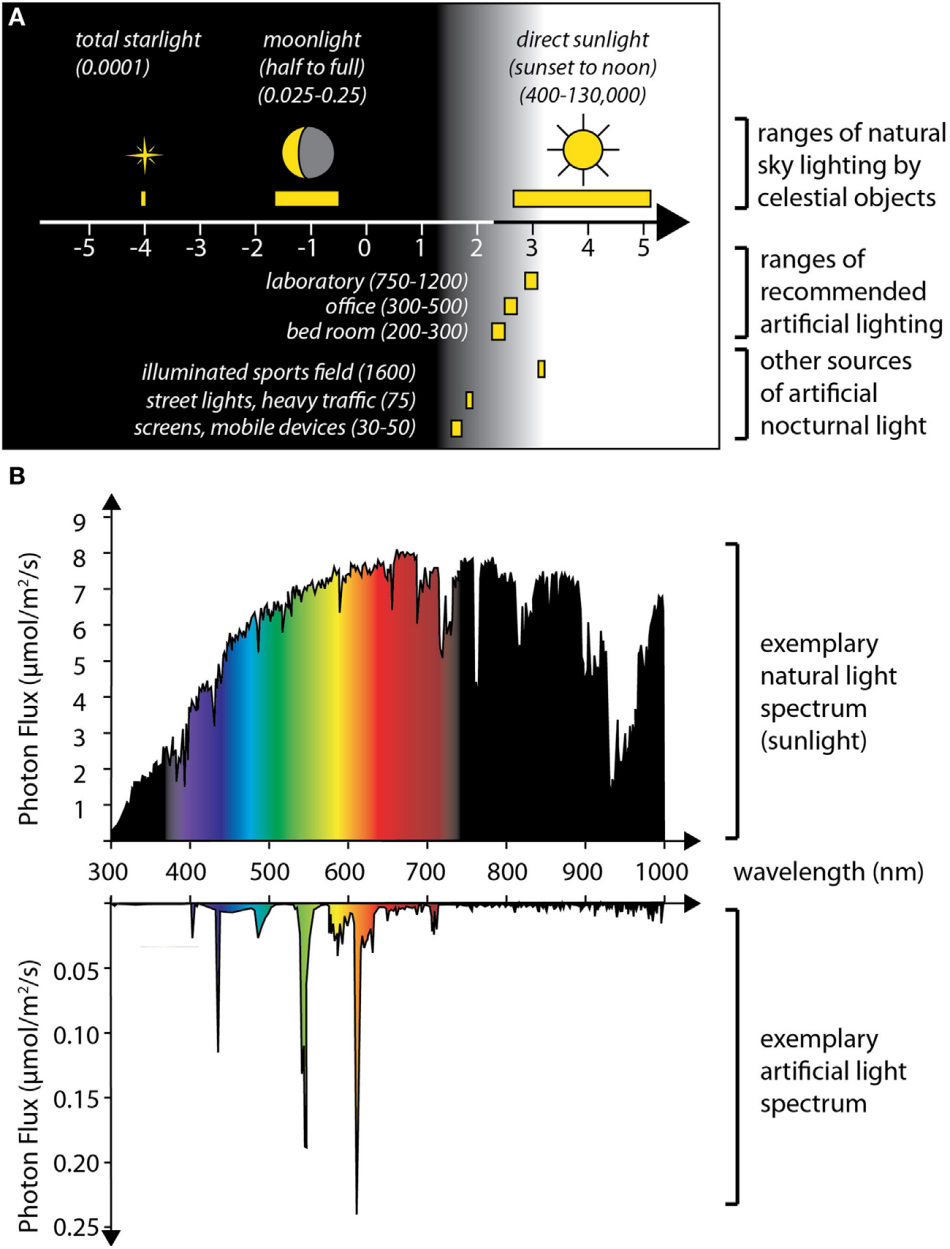
**Levels and spectra of artificial light compared to natural light sources**. **(A)** The graph summarizes published values on the illuminance caused by celestial bodies (up) (top), and of various sources of artificial light (bottom), expressed in lux, plotted on a logarithmic scale. All displayed artificial light sources cause intensities that exceed maximal moon light intensities (approximately 0.25 lx at a clear full moon night) by at least two orders of magnitude. This indicates that artificial light is highly likely to interfere with any natural response to moonlight. **(B)** In addition to light intensities, artificial lights also have various distinct spectra. Top: Photon flux (expressed as micromoles per square meter per second photons) across the light spectrum (in nanometers), measured for sunlight (on noon of a summer’s day, Vienna, Austria); bottom: spectrum of a Philips compact fluorescent lamp (14 W); depending on the specific light receptors affected, the effect of artificial light at night can be aggravated or reduced by changing its spectral composition. Data in panel **(A)** compiled from Ref. (52, 53) and an online version of the Handbook published by the Illuminating Engineering Society (https://www.archtoolbox.com/materials-systems/electrical/recommended-lighting-levels-in-buildings.html); spectra in panel **(B)** schematized based on published values (54).

Besides the search for moon light sensors, another interesting aspect is the actual mechanism by which organisms distinguish moonlight from other light. One specific question is if there are mechanistic parallels, or even deeper evolutionary links, between the detection of nocturnal light stimuli (relevant for circalunar rhythms or circalunar clock entrainment) and the detection of long vs. short photoperiods (relevant for seasonal rhythms and the entrainment of circannual clocks). Photoperiodism is a widespread phenomenon, occurring in phyla ranging from rotifers and annelids to birds and mammals, helping these animals to anticipate the change of seasons and to adjust their physiology and behavior accordingly [reviewed in Ref. (50, 51)]. There are interesting commonalities between the detection of long photoperiod and moonlight: (i) in both cases, the relevant light stimulus is weaker than the sunlight that animals are exposed to during the day: the setting or rising sun causes less than 1% of the illuminance of the sun at noon. For moonlight, this difference in light intensity is even around five to six orders of magnitude (see Figure 2). (ii) Even though the intensity of the relevant light stimulus is therefore very small, in both cases, the actual time point of the stimulus with respect to the 24-h cycle is highly relevant for its interpretation.

For photoperiodic light detection, these considerations have led to the proposal of a “coincidence model,” whereby the circadian clock of an organism allows it to set a certain time window of sensitivity, in which the presence vs. absence of light—even if weak—can be correctly interpreted as indication of long- vs. short-day length (18, 55). Interestingly, molecular analyses in mammals have revealed a gene regulatory system that matches this coincidence model. In the sheep pars tuberalis, transcript levels of the transcription factor Eyes absent 3 (Eya3) are controlled both by a circadian signal (that licenses *eya3* transcription 12 h after night fall) and by the acute levels of melatonin (that lead to a suppression of *eya3* transcription during darkness). As melatonin levels are suppressed by light, the combination of these regulatory mechanisms leads to a specific upregulation of *eya3* only under short photoperiod (56). Melatonin-proficient mice appear to possess a similar ability to induce *eya3*, suggesting that this mechanism could be evolutionarily conserved (57).

Given the very low intensity of moonlight, the coincidence model is also one plausible model how a moonlight stimulus could be detected by animals and distinguished from daylight. Like in the case of the photoperiod, the lunar cycle leads to periodic changes not only in the intensity but also the time of nocturnal light (as moon rise and moon set move with respect to the circadian cycle). These features could allow an animal to detect a change in lunar phase by a switch in light state during a sensitive nocturnal period. Experimental data in the midge *Clunio* are compatible with such a model (58); likewise, in *Platynereis*, the relevant stimulus for circalunar synchronization appears to be that the animals obtain a switch from a “light on” state to a “light off” state; notably, this could even be a switch between a long-day photoperiod to a short-day photoperiod, providing a direct parallel to photoperiodic responses in other animals (11). To which extent such mechanistic parallels might also be reflected in molecular similarities is still far from clear. It is interesting, however, that in one of the aforementioned studies on coral gene expression, Brady et al. also observed that levels of a gene with similarity to the *eyes absent* family changed when compared between full moon and new moon nights (23). Moreover, the mentioned influence of the lunar cycle on melatonin signaling in fish provides another interesting molecular link that might help to delineate similarities and differences between moonlight reception and photoperiodic mechanisms.

## Relevance for Terrestrial Species

As outlined earlier, the presence of circalunar rhythms and clocks across a broad spectrum of marine species (see Figure 1) is consistent with the idea that the respective timing mechanisms already predate the major diversifications of animals and the conquest of land. This would imply that also the ancestors of land-living animals likely possessed similar mechanisms. If so, is there evidence for any remnants of these mechanisms in land-living animals, including mammals? In keeping with the distinction between direct environmental impact and clock-mediated processes that we referred to above, we will focus here on two different aspects: the impact of nocturnal light or gravitational cycles on the physiology of terrestrial animals, and the evidence for internal clocks with a monthly period.

### Influence of the Moon on Reproductive Timing of Terrestrial Animals

Given the strong role that the moon plays in popular belief and human mythology, scientists have generally remained critical toward reports of direct lunar impact on humans or other terrestrial animals. Moreover, light pollution caused by the process of industrialization/electrification is a factor that is likely to obscure natural responses to moonlight or even disturb the respective rhythms (see the more extended discussion on this topic below).

Nonetheless, a series of scientific studies has produced evidence for the existence of circalunar or circasemilunar rhythms also in terrestrial species. These affect diverse animal phyla and various aspects of animal life, ranging from reproduction to communication or behavior related to preying or protection from predators [reviewed in Ref. (59)]. Here, we focus primarily on reproductive rhythms, as these allow to consider differences and similarities to the reproductive rhythms introduced in the above sections.

One case where reproductive timing appears to be linked to the lunar cycle is the Serengeti wildebeest, a grazer that migrates each year in herds of enormous size across the Serengeti. The calves of the Wildebeest are typically born in a very narrow, 3-week period around January or February, months before the mass migration in May/June (60). One likely advantage for the synchronized reproduction is that it reduces predation risk. Using birth dates as well as embryo sizes, conception dates in this animal have been systematically estimated. Even though exact estimates are not possible (60, 61), it is remarkable that the estimated dates—which vary from year to year on the solar calendar—consistently fall into a time window in April to May that is determined by two consecutive full moons (60). It thus appears that—on top of a seasonal signal—the fine tuning of conceptions is linked to the lunar phase. Of note, in the equatorial region, the waning and waxing moon probably provides a more robust light cue than the small differences in solar timing. Hence, other terrestrial animals in the equatorial zone that display narrow reproductive peaks may be interesting species to search for lunar reproductive mechanisms.

Another example of a mammal where the time window for conception appears to be correlated with the moon phase is the European badger. Also in this species, lunar timing is superimposed on a seasonal breeding cycle. The animals typically mate in February to March, soon after the females have given birth to the previous litter. During lactation, the embryos are in a diapause state, before seasonal cues (photoperiod and temperature) lead to implantation around the end of December (62, 63). Dixon and colleagues performed a systematic, long-term video surveillance study on badger behavior during the mating season and also compiled published records from over 100 years of natural history literature on badger behavior (64). The study then investigated the exact dates at which animals copulated and also tracked stereotypic behavior associated with mating, such as the increase in territorial behavior, as evidenced by squat marking (in both sexes) and raised-leg urination (in males). For all of these behaviors, the authors observed a significant correlation to the moon phase, with a peak around the new moon phase.

The third, less pronounced, but surprising case linking the moon phase to mammalian reproduction has been reported for domesticated cattle. In a systematic, 3-year study on over 400 Holstein cows raised on a Japanese farm, Yonezawa and colleagues recently reported a significant influence of the moon phase on spontaneous delivery dates, with deliveries peaking shortly before the full moon, while being minimal around new moon (65). As the animals were artificially inseminated, and the insemination dates were accurately recorded for each cow, the authors were able to show that the observed pattern was not generated by a fluctuation of conception frequencies. Rather, they could relate the observed pattern to deviations between the expected and actual delivery dates. Specifically, there was a significant effect of the moon phase in delaying (new moon) or accelerating (full moon to waning gibbous phase) the actual delivery for up to 2 days (65). The resulting changes are less than 1% in gestation length (average: 284 days), but the study strongly suggests that in a well-controlled system (low genetic variation, reduced artificial light sources), physiologically relevant effects of moonlight can be determined. Given that cattle have been domesticated for around 10,000 years (66), it is possible that this effect represents just a remnant of a more pronounced trait that might have been more relevant in the wild.

When comparing these examples with the aforementioned reproductive cycles in many marine animals, two aspects are interesting to note: (i) in terms of reproductive strategies, mammals are characterized by internal fertilization and typically an independence of the tides. This strategy represents an obvious contrast to the marine broadcast spawners and species reproducing within the tidal zone, where precise synchronization of mating time between sexes is essential for maintaining reproductive success. Otherwise, germ products would be quickly diluted in the water or the substrate/niche required for egg deposition would be unavailable. By contrast, internally fertilizing animals can uncouple copulation from fertilization. This happens, for instance, in numerous insects, with storage of sperm for periods of days to months or even years. Likewise, as demonstrated by the example of badgers, internally fertilizing animals can also uncouple fertilization from embryonic development if needed. Therefore, if circalunar control of reproduction was indeed a more ancient feature of reproduction, the selective pressure to maintain it would have become more relaxed in species evolving internal fertilization strategies. Other selective advantages might therefore be more relevant for the maintenance/evolution of circalunar reproductive strategies in such lineages, for instance, the ability to limit the chance for predators to prey on the offspring (wildebeest). (ii) The two highlighted species in which copulations are limited (wildebeest and badgers) display a clear seasonality in reproduction. The lunar cycle therefore is not the only relevant cycle governing reproduction but is also integrated with information on the season. It is still unclear if these species use light cues to derive information on both lunar phase and seasonal state, or if other cues (temperature for the season; gravity for the lunar phase) may play a role.

### Effects of the Moon on Human Birth Rates?

Classical authors as well as popular mythology also suggest various effects of the moon on human biology. These range from an influence on the menstrual cycle and birth dates to aggressive behavior or an impact on mental health [reviewed in Ref. (67)]. Any of these effects is discussed in a controversial manner. Here, we will mainly review three of these aspects: the question if the moon has an influence on human birth rate, the question if there is a connection between the lunar cycle and sleep, and the question if the lunar cycle affects mental health.

Concerning reproduction, one popular claim is that human births are not randomly distributed over the month, but that birth rates differ over the course of the lunar cycle. As for the cases of animal reproduction mentioned earlier, scientists have begun to systematically analyze such claims in the twentieth century. One of the first studies systematically investigating the frequency of births in a large, longitudinal study (1948–1957, around 250,000 births) concluded that around the full moon, birth rates (calculated as a sliding window of 3-day averages) were between 2 and 3% elevated over the average, whereas the time point around new moon showed a reduced birth rate (2–3% below average). The effect was found to be statistically significant (68) and was also consistent with a subsequent study that was conducted for around 500,000 births over a shorter period (1961–1963) (69). Interestingly, the described differences match well with the differences reported by the aforementioned study by Yonezawa and colleagues in the parturition of cows (65). In the decades to follow, various studies have investigated the correlation between birth rates and lunar phases in independent, and partly larger, datasets. Results, however, varied: some found support for an influence of the lunar phase on birth rates, such as a study by Guillon and colleagues on more than 12 mio births in France between 1968 and 1982 that confirmed a local maximum around full moons (70) (in addition to non-random distributions around quarter moons). Others, however, do not find evidence for a significant correlation, such as a study by Waldhoer and colleagues on around 2.5 mio births in Austria between 1970 and 1999 (71). One way to interpret these inconsistencies is that there is no real influence of the moon on human births, that earlier studies are to be dismissed as outliers, and/or that their methodology underestimated false positive rates (67, 71). On the other hand, it is worth to consider that there could also be anthropogenic factors that introduce biases, especially in more modern datasets. Menaker and Menaker already commented that they excluded data from private clinics, because they displayed obvious drops in births correlating with weekends (especially Sundays). The authors attributed this to the reduced inclination of private doctors to come in on these days (68). Medical development over the following decades introduced various ways in which deliveries could be artificially induced, for instance, by oxytocin or prostaglandin treatment, or amniotomy. Moreover, the frequency of caesarian sections has increased in many countries, now ranging around 30% in the US, Germany, or Austria, and even higher rates in middle and South America, peaking at more than 50% in Brazil (72). Obviously, any of these techniques offers the possibility to induce birth before the natural date and therefore represent factors that would obscure any small effect on natural birth dates caused by the moon at modern times. Of note, both of the more recent studies report a significant drop of birth rates on weekends in their respective datasets (70, 71).

### Possible Lunar Effects on Mental Health and Sleep, and the Role of Artificial Nocturnal Light

Another area that has attracted significant interest is the question if the lunar cycle has any impact on mental state of humans. A connection between the moon and mental health is deeply rooted in etymology: the Latin word for moon (luna) is contained in the German word “Laune” (=mood); likewise, the Oxford Dictionary explains that the old Latin word “lunaticus” gave rise to French “lunatique” or English “lunatic,” with the word “monseoc” (“moon-sick”) representing an old English equivalent to this term. All of these terms relate to the concept that certain persons exhibit periodic phases of mental illness or mood swings, with the earliest use likely relating to epileptic episodes (73, 74). The question is if the link to the moon represents a mere analogy or mythological connection, or if it reflects a—direct or indirect—influence of the moon on mental states.

Different authors have provided alternative explanations for the origin of this connection, and on the role nocturnal light might play in that context: one line of arguments is that in the pre-industrialized world, moonlit nights—especially the three days surrounding full moon—provided a natural opportunity to perform work, hunt, or travel (73), and that these nights therefore led to a decrease in human night sleep around the full moon until around 200 years ago. As reduced sleep is a common parameter in conditions causing mania in patients with bipolar disorder (75) and can also increase the chance of epileptic seizures (76, 77), such monthly recurring phases of reduced sleep might form the factual core of the popular association between the lunar phase and mental health (67, 73). Following this line of reasoning, the advent of artificial illumination ended the dependence of humans on moon light as an exclusive nocturnal light source, thereby causing more stable sleep–wake patterns, and dissolving any apparent lunar periodicity in mental illnesses. In line with this, modern studies do not find a connection between epileptic seizures and the full moon (78).

While this explanation emphasizes the secondary nature of sleep deprivation (as a consequence of a cultural habit to work on moonlit nights), other authors suggest that the lunar cycle itself has a relevant effect on sleep, thereby reflecting a more direct impact of the moon on human physiology. Whereas this issue still remains controversial, two concepts need to be distinguished. On the one hand, the lunar cycle—for instance, the difference in light intensity—might directly impact on sleep parameters. This is, for instance, consistent with a large study on children in 12 different countries whose activity patterns were monitored by accelerometers. This study reported a significant shortening of sleep around full moon by about 5 min (79). The children in this study were monitored remotely in their home environments, and thereby could have been exposed to moonlight in their bedrooms.

Most studies on that subject, however, result from sleep laboratories, in which external light sources like moonlight were systematically excluded. Surprisingly, even under such conditions, effects of the lunar cycle on human sleep have been reported: Cajochen et al. reported a correlation between human sleep quality and the state of the moon in a dataset comprising sleep recordings from 33 subjects, with deep sleep patterns (−30%) and total sleep time (−20 min) being significantly reduced around full moon (80). As the authors emphasized, the analysis was performed only *post hoc*, such that neither the subjects nor the scientists involved in the original experiments could have been biased. Independent studies also arrived at the conclusion that the lunar phase affected sleep, while differing in the detail: consistent with the study by Cajochen et al., Smith et al. reported a reduction of total sleep time during full moon in a study focusing on 47 volunteers, but reported that this overall effect was driven by the sleep patterns of men (−50 min), pointing at possible differences in sex, at least for young subjects (see below). By contrast, Turányi and colleagues, focusing on patients with sleep disorders, reported a stronger effect on women (81). Likewise, a study by Della Monica et al. on 205 healthy subjects, arrived at the conclusion that women had a significantly reduced total sleep time during full moon, whereas men in this study showed even an increase, such that the net effect (irrespective of sex) was not significant (82). The interpretation of differences in these studies varies: some take them as evidence that effects of the lunar phase on sleep exist, but may vary depending on sex or age. For instance, women in the Della Monica study were primarily postmenopausal, whereas in the study by Smith et al., the individuals were on average 23 years old (82, 83). Such differences could also explain why significant net effects were not observed in a re-analysis of three large sleep datasets (covering together more than 2,000 individuals, not separated by sex) by Cordi and colleagues (84). Conversely, as for the discussion of human birth rates, the argument has been made that smaller datasets might produce significant correlations merely by chance, and that non-significant results are unlikely to be published, generating a confirmation bias in the published—and publishable record—on that matter (82, 84).

Adding to this discussion, the third possibility also exists: if one accepts the idea that endogenous circalunar clocks could also exist in humans (see below), and that they impacted on sleep structure, it would currently be completely unclear if the subjects in either of the mentioned studies were likely to have synchronized oscillations or if these oscillations were offset between individuals. For instance, if nocturnal light played a role in adjusting an individual’s circalunar clock—as evident for several of the marine species discussed earlier—changes in the availability and spectrum of nocturnal light could easily impact on the phase of such a clock. Remarkably, artificial light sources have begun to dramatically change the natural fluctuations of light conditions that organisms have experienced during their evolution (52) and are changing both the spectrum and intensity of nocturnal light, with local increases in intensities of up to 20% per year (85). A comparison of illuminance levels shows that recommended levels for room lights, as well as the illuminance from traffic or mobile phones, far exceed the illuminance even of a bright full moon (Figure 2). Moreover, the spectral composition of artificial light sources can strongly deviate from sun or moon light, and blue components in artificial lighting are already known to affect melatonin production and animal circadian clocks, even though the natural entrainment stimulus for these is orders of magnitude more intense (Figure 2). Given these considerations, it is clear that the impact of artificial lights on biological clocks or rhythms responsive to moonlight levels could be even more dramatic. In turn, this makes the identification of physiological effects of moonlight on human subjects inherently difficult at modern times.

Whereas the majority of the discussed arguments considers moonlight as the most likely cue that impacts on animal physiology—either directly or *via* circalunar clocks—a recent publication by Wehr (86) argues in favor of another possibility. By analyzing a set of longitudinal studies (up to 7 years) in patients with bipolar disorder, the author found evidence that episodes of rapid switches between mental states (mania to depression or *vice versa*) did not occur randomly. Rather, these episodes—as well as some pronounced switches in their frequency—were coupled to gravitational cycles of the moon. For example, when the author compared the mood cycles of patients with the 14.8-day cycle that characterizes the re-occurrence of the axis of moon, sun, and earth (spring-neap tidal cycle), these cycles had particular phase relationships. In some patients, a complete mood cycle (e.g., depression—mania—depression) occurred every two biweekly lunar cycles (i.e., every lunar month, 29.5 days). In other cases, there seemed to be a resonance between one mood cycle and three biweekly lunar cycles (44.3 days), or other integer relationships (86). Moreover, when the author assessed when switches in these periodic relationships occurred (for instance, major shifts in frequency from shorter mood cycles to longer mood cycles), these repeatedly coincided with the 206-day recurrence of the perigee-syzygy constellation of the Earth–Moon–Sun system (“supermoon”) which is marked by the coincidence of a full moon with the closest proximity of the moon on its elliptical orbit around the earth. While these constellations also represent an increase in full moon illuminance by about 30%—a factor that ought to be taken into account by studies on moon light effects as well (87)—the favored interpretation by the author is that the coincidence with mood switches in the patients is caused by some gravitational influence, even though the nature of this effect would currently remain unclear (86).

### Circalunar Clocks in Mammals

In summary, the above examples illustrate that there are various indications for an influence of the moon on the physiology of humans and other mammals, and point toward aspects that require more detailed analyses. As to humans, several authors have emphasized the need for more extensive longitudinal studies that could better resolve inter-individual differences [see, e.g., Ref. (82)]. Likewise, light pollution will need to be taken into consideration for both human and animal studies, especially as this phenomenon is increasing on a global scale (85), not only affecting terrestrial but also marine environments (88). As pointed out, light pollution is at least a potential caveat when scientists dismiss early studies on circalunar rhythms or clock phenomena based on more recent studies—which is not to say that older analyses could not have failed.

Controversies about the extent of lunar influence, however, should not distract from another physiological phenomenon that is worth emphasizing at the end of this review: primates including humans clearly possess hormonal cycles with monthly periodicity, indicating that there must be timekeeping mechanisms in humans as well as other mammals that are able to run with a roughly monthly period. The most prominent cycle is the menstrual cycle of women who has been determined to cycle almost precisely with a lunar monthly period (29.5 days) (68). Recent data from human males who were kept isolated in a highly controlled environment indicates that such hormonal cycles are not limited to females, but can occur in males as well (89), arguing that the respective timing mechanisms are general properties of human biology. Also outside primates, estrous cycles with a period length of around a month exist. For instance, the estrous cycle of badgers—mentioned above for the role of the lunar phase on the animals’ conception dates—has been reported to take approximately one lunar month (90).

Of course, any of these correlations might be coincidence. But one alternative speculation is that such cycles could also be the remnants of an ancient situation where clocks with a monthly period were indeed synchronized by external factors (such as gravity or nocturnal light). A likely scenario then was that the relevance of that synchronization was secondarily diminished—for instance, by a shift in selective pressure that reduced the advantage of a synchronized reproductive window. As a consequence, the endogenous clocks might subsequently have been uncoupled from their respective entrainment mechanisms, while still persisting as oscillators that structure the timing of mammalian physiology. This scenario does not exclude that there could be a remnant capacity of hormonal cycles to be entrained by nocturnal light, as has been suggested by some experiments for the human estrous cycle [reviewed in Ref. (20)]. But rather than putting emphasis on such remnant capacities, this hypothesis would make the prediction that the actual timing mechanisms between mammals and non-mammalian animals with circalunar clocks share ancient commonalities.

## Author Contributions

FR and KT-R conceived and wrote most of the manuscript. HT contributed specific subsections and also provided constructive feedback.

## Conflict of Interest Statement

The authors declare that the research was conducted in the absence of any commercial or financial relationships that could be construed as a potential conflict of interest.
